# Contagious yawning and scratching in captive lemurs

**DOI:** 10.1038/s41598-024-77805-9

**Published:** 2024-11-04

**Authors:** William Padilha Lemes, Federica Amici

**Affiliations:** 1 Department of Evolutionary Zoology and Human Biology, University of Debrecen, Debrecen, Hungary; 2 Faculty of Life Sciences, Institute of Biology, Human Biology & Primate Cognition, Leipzig University, Leipzig, Germany; 3 Department of Comparative Cultural Psychology, Max Planck Institute for Evolutionary Anthropology, Leipzig, Germany

**Keywords:** Ruffed lemurs, Strepsirrhines, Emotion, Primates, Attentional bias hypothesis, Ecology, Evolution

## Abstract

Behavioral contagion is thought to play a significant role in social synchronization and coordination across animal taxa. While there is extensive evidence of behavioral contagion in Haplorrhines (i.e. monkeys and apes), limited research exists in Strepsirrhines (i.e. lemurs). Here, we aimed to investigate the presence of contagious yawning and scratching in two captive groups of black-and-white ruffed lemurs (*Varecia variegata*) (*N* = 4) and red ruffed lemurs (*Varecia rubra*) (*N* = 4), and further test whether behavioral contagion is modulated by the model’s social integration in the group. We conducted all occurrence sampling to examine whether individuals observing a yawning or scratching event (i.e. trigger event) were more likely to yawn or scratch in the following 2 min, as compared to individuals who did not observe it. We ran generalized linear mixed models and found that the likelihood of yawning and scratching was higher for subjects observing the trigger event than for subjects who did not observe the event, although the model’s social integration had no modulating effect on the probability of showing behavioral contagion. Our findings represent the first evidence of behavioral contagion in this genus and contribute to shed light on the distribution and the possible adaptive function of this phenomenon in primates.

**Supplementary Information:**

The online version contains supplementary material available at 10.1038/s41598-024-77805-9.

## Introduction

Behavioral contagion is the propensity to show a behavior (e.g. yawning or scratching) after observing or hearing it being displayed (i.e. trigger event) by another individual^1,2^. From a functional perspective, behavioral contagion might increase individual fitness by facilitating individuals’ synchronization within dyads and groups^3^. Such synchronization may be adaptive by, for instance, increasing social cohesion (e.g. individuals are active or inactive at the same time), decreasing offspring mortality through synchronized reproduction (e.g. reducing infanticide risk and predation pressure on offspring), and generally enhancing the effectiveness of anti-predator strategies (e.g. through collective group movements that reduce individual predation risk and increase the synchronization of vigilance bouts)^4^.

Most studies on behavioral contagion have focused on yawning and partially on scratching, because these behaviors are relatively easy to recognize during observational studies and can provide important information about the mechanisms that allow behaviors to spread within animal groups^5–7^. Spontaneous yawning and spontaneous scratching are both fixed action patterns that are relatively widespread among vertebrates: while spontaneous yawning has a strong physiological component^8^, and its occurrence can increase when individuals are aroused or need to facilitate thermoregulation and brain oxygenation^5,9^, spontaneous scratching is the mechanical result of the unpleasant itch sensation^10^, and can be influenced by both physical and psychological factors^11^. In contrast, the behavioral contagion of these behaviors might not be as widespread. According to some researchers, it might have only recently emerged in vertebrate evolution, in highly social species^12,13^, as it may be linked to emotional arousal and have a largely communicative function^14^. Although initial studies thus focused on behavioral contagion in group living animals with high social complexity (rather than pair-living or solitary species), more recent studies have challenged this hypothesis by providing evidence of behavioral contagion also in species with lower social complexity, like semi-solitary ones^3,15^. Indeed, it is possible that behavioral contagion does not only or primarily serve a social function, as usually assumed, but rather constitutes a bottom-up mechanism that facilitates the prediction of environmental cues, and can be highly adaptive even if the opportunities to rely on it are relatively scant^16^.

Studies on contagious yawning have found evidence that, in several species, observing a model yawning increases the probability that individuals will also yawn^1,17^. Evidence of contagious yawning has mainly been provided in species living in complex social groups (e.g. often engaging in cooperative interactions^13^, being highly prosocial^14^, showing high levels of fission-fusion dynamics^18^), such as social birds like budgerigars (*Melopsittacus undulates*)^19^, and mammals like sheep (*Ovis aries*)^20^, wolves (*Canis lupus*)^21^, dogs (*Canis lupus familiaris*)^22^, elephant seals (*Mirouga leonina*), pigs (*Sus scrofa*)^17^, lions (*Panther leo*)^11^ and African elephants (*Loxodonta africana*)^24^. Among primates, studies have primarily focused on Catarrhines. For instance, contagious yawning has been observed in orangutans (*Pongo* spp.)^25^, bonobos (*Pan paniscus*)^14,26^, chimpanzees (*Pan troglodytes*)^27,28^, stump-tailed macaques (*Macaca arctoides*)^29^, wild geladas (*Theropithecus gelada*)^30^ and humans (*Homo sapiens*)^31^, although there is yet no evidence of contagious yawning in lowland gorillas (*Gorilla gorilla gorilla*)^32^. More recently, however, contagious yawning has also been shown in primates other than Catarrhines, including Platyrrhines (i.e. spider monkeys, *Ateles geoffroyi*)^15^ and Strepsirrhines (i.e. indri lemurs, *Indri indri*)^3^, where behavioral contagion was thought to be absent. In the first study on lemurs, Reddy et al.^13^ detected no contagious yawning in ring-tailed lemurs (*Lemur catta*) and red ruffed lemurs (*Varecia rubra*) when using video stimuli as trigger events, and suggested that contagious yawning might have evolved in the common ancestor of Catarrhines and Platyrrhines after the lineage split from Strepsirrhines. However, Valente et al.^3^ recently found evidence of contagious yawning in wild indri lemurs, with individuals being more likely to yawn after observing a trigger event.

Studies on contagious behavior have shown that scratching can also be contagious^33^. In humans, for instance, scratching can be triggered by hearing the word “itching” or the sound associated with itching, as well as by witnessing others scratching^34–36^. Previous studies have shown that videos or pictures of people scratching can induce scratching behavior in healthy individuals^34^. Besides humans, contagious scratching has been observed in a few other species, including rhesus macaques (*Macaca mulatta*)^33^, Japanese monkeys (*M. fuscata*)^37^, Tibetan macaques (*M. thibetana*)^38^, orangutans^39^, spider monkeys and mice^40^. In contrast, a study in common marmosets (*Callithrix jacchus*) has failed to detect contagious scratching^41^, and there are so far no studies on contagious scratching in Strepsirrhines.

Several studies have also identified factors that might modulate behavioral contagion. For some authors, behavioral contagion is linked to empathy and to the ability to align to others’ internal states, and it may thus be higher between kin or individuals that have close social bonds^12,13,25,28,42^. In line with this, humans are more likely to yawn after close friends or family members yawn^43^, and bonobos and geladas are more likely to yawn when triggers are kin or close social partners^14,44^. Similarly, in wild *Indri indri*, individuals that more often groom each other are also more likely to show contagious yawning^3^, and chimpanzees are more likely to yawn after observing yawning by group members rather than outgroup members^28^. Other authors, however, contend that behavioral contagion is more frequent among close friends and family members because of higher selective attention paid to individuals that are more relevant to the observer (attentional bias hypothesis)^12,42^. If this is true, behavioral contagion should be more frequent not only when models are kin or close social partners, but also when they play a relevant social function in the group (i.e. highly ranking or socially integrated group members). In chimpanzees, for instance, there is evidence that male models are more likely to trigger contagious yawning than females, especially when they are dominant^43^.

In this study, we aimed to investigate the presence of behavioral contagion in two species of ruffed lemurs: one captive group of black-and-white ruffed lemurs (*Varecia variegata*) and one of red ruffed lemurs (*Varecia rubra*). In contrast to other Strepsirrhines species (e.g. *Mirza coquereli*, *Lepilemur sahamalazensis*), which are typically described as ^47,48^, ruffed lemurs are characterized by complex sociality (i.e. high levels of fission-fusion dynamics, alarm/call synchronization, cooperative nest-sharing, communally infant care)^49–52^ and thus constitute an ideal model to study behavioral contagion. We predicted that, as in the study on wild Indri^3^, individuals would be more likely to yawn or scratch after observing a conspecific yawning or scratching, rather than if they did not observe such trigger event (Prediction 1). Furthermore, according to the attentional bias hypothesis, socially integrated individuals may be more relevant to the observers^42^ than loosely integrated ones, and thus more likely to trigger behavioral contagion. Therefore, we predicted that behavioral contagion would be more likely triggered by models that were highly rather than loosely integrated in their group (Prediction 2). Although model’s rank might also affect the model’s relevance to the observers and thus mediate the probability of triggering behavioral contagion^42^, we decided not to test this hypothesis because our study groups only included one mature couple each, so that information on the individuals’ rank was not really reliable. Similarly, we did not test the modulating effect of kinship^12,14,42^, because our study group only included kin.

## Methods

### Study site and study subjects

This study was carried out at the Nyíregyházi Állatpark Nonprofit Kft (Sosto Zoo), located in Nyíregyházi, Hungary. Study subjects included one group of black-and-white ruffed lemurs (*N* = 4) and one group of red ruffed lemurs (*N* = 4). Individuals could be individually recognized through their distinct features, such as unique color patterns in their backs and hind limbs. The group size and composition remained constant throughout the study. Within both groups, all the individuals were kin and included two offspring, their mother and father. The black-and-white ruffed lemur group included 1 male and 3 females (Table 1). During the summer, the group was housed in an outdoor enclosure (6 m wide × 9 m long × 3 m high); in the winter, they were moved to an indoor enclosure (5.3 m wide × 4 m long × 3.5 m high). The red ruffed lemur group included 2 males and 2 females (Table 1). This group was housed in a mixed enclosure with an outdoor area (7.3 m wide × 13 m long × 3 m high) and an indoor area (1.5 m wide × 5.5 m long × 2 m high) that they could use both in summer and winter. The indoor area was shared with a group of black lemurs (*Eulemur macaco*) and ring-tailed lemurs. Demographic information on the group members (i.e. age, sex, kinship) was provided by the Zoo.

**Table 1:** For each species and individual, demographic (i.e. sex, age, relationship) and social (i.e. rank and Eigenvector centrality, as a measure of social integration) information.

Species	Identification	Sex	Age (years)	Relationship	Centrality
*Varecia variegata*	Pbw	Male	20	Parent	0.439
	M	Female	17	Parent	0.888
	F1	Female	1	Offspring	1
	F2	Female	1	Offspring	0.916
*Varecia rubra*	C	Male	19	Parent	0.882
	Pred	Female	18	Parent	0.732
	A	Female	2	Offspring	0.700
	N	Male	3	Offspring	1

### Ethics statement

This research was entirely observational and required no manipulation of the study individuals and no restriction of food or water. Individuals were already habituated since many years to the presence of human observers (zoo visitors and zookeepers) right outside their enclosures. The permit to conduct the research was provided by the Nyíregyházi Állatpark Nonprofit Kft. (Sosto Zoo) where the subjects were housed. Our approach adhered to the ethical principles of the American Society of Primatologists for the treatment of nonhuman primates, and to the Code of Best Practices for Field Primatology as published by the same society.

### Data collection

We collected data on yawning and scratching events from August 2023 to January 2024, from 08:00 am to 05:00 pm, for a total of 243 h of observation (i.e. 166 h for black-and-white ruffed lemurs and 77 h for red ruffed lemurs). Before starting data collection, the observer (i.e. the first author) underwent a 3-week period of training to familiarize himself with the individuals and the methods. The observations were conducted in 15-minute blocks and alternated with 5-minute breaks to provide rest to the observer, who recorded the data on paper sheet. Following previous studies^3,15,44^, we recorded all occurrences of yawning and scratching only in the absence of perturbing factors (i.e. we did not conduct observations when zookeepers were inside the enclosure for daily cleaning and feeding routines, when visitors were in front of the enclosure within the group’s view, and when group members produced alarm calls, for the whole duration of these events), to reduce the number of yawning and scratching events triggered by external factors^53^. We chose the all-occurrence method due to the low number of subjects in each group and the high visibility offered by the enclosures, which allowed us to reliably observe all the yawning and scratching events in the group, as well as the response of the other group members.

Whenever an event of yawning (i.e. one individual engaged in deep inspiration, followed by a lengthy, forceful expiration, with simultaneous contraction of many skeletal muscles) or scratching (i.e. one individual repetitively drew its nails on its own skin with the fingertips) occurred in the group (hereafter, trigger event), we recorded the following information: (a) the identity of the model performing the trigger event; (b) the type of trigger event (i.e. yawning or scratching); (c) the distance between the model and the other group members (hereafter, subjects) when the trigger event took place (i.e. body contact, < 1 m, 1–2 m, > 2 m); (d) which subjects could see the trigger event, as assessed based on their facial orientation (i.e. subjects were considered to see the trigger event if they were looking toward the model’s face, or if their face was turned up to a 45-degree angle); (e) whether each of the subjects yawned/scratched within 2 min from the corresponding trigger event. We selected a 2-min time window to allow the comparison with previous studies on other primate species (including lemurs^3,13^, spider monkeys^15^, bonobos^14^, baboons^44^ and orangutans^25,39^, where the time window after the trigger event varied between 90 s and 5 min). This choice was further based on the results of previous studies showing that behavioral contagion in primates usually peaks within the first two minutes from the trigger event^30,38^ (e.g. in spider monkeys, the mean ± SD latency between the trigger event and the partner’s contagious response was 62 ± 53 s for partners observing a yawning, and 32 ± 28 s for partners observing a scratching^15^). We considered every yawning displayed as a single event, whereas for scratching we considered a new event every time a scratching bout was interrupted for more than 3 s^15^.

Finally, we conducted hourly scans to record the spatial distribution of all the group members (i.e. which individuals were within arm reach from each other)^54^, selecting a 1-hour interval to increase the independence of the single scan observations.

### Data analysis

To determine individuals’ social integration in the group, we first assessed the social network based on the hourly scans^55^ of each group in which we recorded, for each individual, the identity of partners within arm reach. We then built an undirected weighted matrix and ran social network analyses with the ‘igraph’^56^, vegan^57^ and asnipe^58^ packages to assess individuals’ Eigenvector centrality (hereafter, centrality) scores^59^, which are the sum of the centralities of an individual’s neighbors and are a measure of the importance of each individual “as a social hub”^55^.

We then ran a Generalized Linear Mixed Model^60^ (GLMM) with the glmmTMB package^61^ in R^62^, using a binomial distribution to evaluate which variables affected the likelihood of yawning and scratching after the trigger event. As response variable, we entered whether the other subjects performed yawning or scratching within the 2 min following the trigger event by the model (0/1). We first ran a full model containing as test predictor the 4-way interaction of the following factors (and their main terms and lower interactions): whether each subject observed or not the trigger event (no/yes), species (black-and-white ruffed lemur or red ruffed lemur), type of behavior displayed in the trigger event (yawning or scratching) and centrality of the model performing the trigger event (from 0 to 1). As control, we further included the distance between the model and each subject (body contact, < 1 m, 1–2 m, > 2 m), and the latter’s sex (female or male). In addition, we entered as random intercepts the identity of the model, the identity of the other subjects, and the identity of the trigger event (as the same trigger event could be observed or not by more than one subject) nested in day identity (as more trigger events could happen on the same day). Given that the full model with the 4-way interaction showed some convergence issues, we proceeded gradually: we first run a full model in which we removed model’s centrality from the interaction (i.e. entering model’s centrality as main term, and the 3-way interaction of whether each subject observed or not the trigger event, species and behavior type, with their main terms and lower interactions); given that the 3-way interaction was not significant, we further simplified it by removing behavior type and entering it as main term in the model (entering instead model’s centrality in the interaction); and we gradually simplified the non-significant interactions, as commonly done in the literature. These models had no convergence issues.

In the majority of cases (i.e. 92%), only up to one subject showed the same behavior as the model after the trigger event; in the other cases, however, more than one subject showed the same behavior as the model, so it is not possible to exclude that the other subjects reacted to this behavior rather than (or as well as) to the original trigger event by the model. Therefore, we decided to run the statistical analyses on the reduced dataset that only included trigger events that were followed by no more than one subject showing the same behavior as the model (*N* = 3162). This criterion, and the choice of a relatively short time window after the trigger event, reduced the probability of autocorrelation in our dataset (i.e. one subject producing the same behavior several times in a row^3,63^).

We checked for multicollinearity in the GLMM using the ‘check_collinearity’ function (R package performance 0.4.4^67^), and found low correlation for all the fixed factors in the model after removing the interactions (max VIF: 1.14). We further used diagnostic plots for hierarchical models (DHARMa package in R^65^) to test assumptions about the distribution of residuals (dispersion = 1.00, *p* = 0.936). For model validation, we compared the final full model to the null model (which only included controls and random factors), using a likelihood ratio test^66^, and then we assessed the significance of the single predictors using the *drop1* function. To estimate the confidence intervals of the model variables, we used the *confint()* function.

## Results

In the study, we observed a total of 170 yawning events. The baseline rate of trigger yawning events produced by the models was 0.15 yawning events per hour, with an increase of 0.11 ± 0.12 yawning events per hour (mean ± SD) when subjects yawned after observing the trigger event, and an increase of 0.02 ± 0.06 yawning events per hour (mean ± SD) when subjects did not observe the trigger event. For the black-and-white ruffed lemur, the probability of yawning was 6.5% after observing the trigger yawning event produced by the model, and 1.3% if not observing it. For the red ruffed lemur, the probability of yawning was 16.6% when observing the trigger event by the model, and 4.4% when not observing it. For scratching, we observed a total of 1650 events. The baseline rate of trigger scratching events produced by the models was 1.07 scratching events per hour, with an increase of 0.35 ± 0.21 scratching events per hour (mean ± SD) when subjects scratched after observing the trigger event, and an increase of 0.15 ± 0.14 scratching events per hour (mean ± SD) when subjects did not observe the trigger event. For the black-and-white ruffed lemur, the probability of scratching was 29.9% when subjects observed the trigger event by the model, and 13.7% when subjects did not observe it. For the red ruffed lemur, the probability of scratching was 18.6% when subjects observed the trigger event by the model, and 8.9% when they did not observe it.

**Fig. 1:**
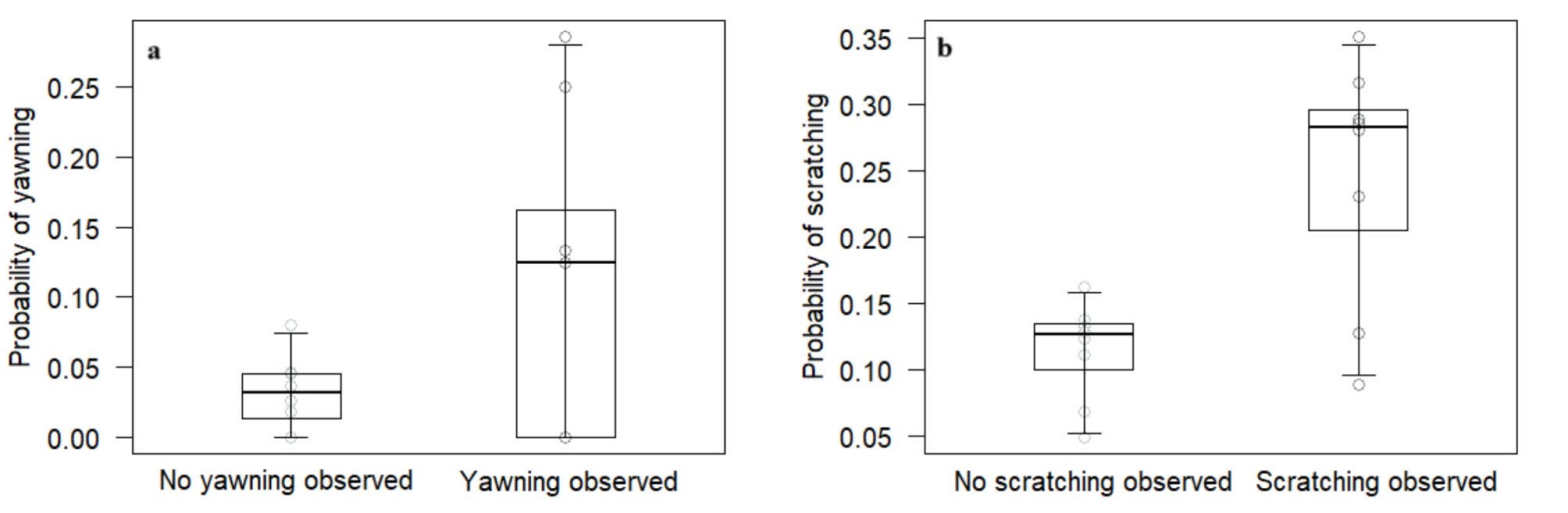
(**a**) Probability that individuals would yawn after observing or not the yawning triggering event, and (**b**) probability that individuals would scratch after observing or not the scratching triggering event. Circles represent average values for each individual. The thick lines represent the median values of the individual means, the horizontal ends of the box represent the 75% and 25% quartiles, and the ends of the whiskers represent the 97.5% and 2.5% quartiles.

The full model was significantly different from the null model (*χ*^2^ = 80.82, df = 4, *p* < 0.001), with several main terms (but no interactions) having a significant effect. In particular, the probability that subjects showed the same behavior as the trigger event was higher when subjects observed the trigger event, as compared to when they did not (*p* < 0.001; Table 2; Fig. 1). Interactions had no significant effect, suggesting no modulating effect of species, behavior type and model’s social integration on the probability of showing behavioral contagion, although both species (*p* = 0.030; Table 2) and behavior (*p* < 0.001; Table 2) were significant as main terms, suggesting that subjects were generally more likely to show the same behavior as in the trigger event (regardless of having observed the trigger event) in black-and-white ruffed lemurs than in red ruffed lemurs, and that they were also generally more likely to scratch than yawn. Additionally, the control variable distance was significant (*p* = 0.010; Table 2), indicating that subjects closer to the trigger event were more likely to show the same behavior displayed by the model, regardless observing it or not.

**Table 2:** For each predictor and control included in the model (after removing the non-significant interactions), mean estimates, standard errors (SE), two-sided 95% confidence intervals (CIs), *z*-values and *p*-values; significant test predictors are in bold, controls in italics and reference categories in parentheses.

Predictors	Estimate	SE	2.5 to 97.5% CI	z value	*p*-value
Intercept	− 2.34	0.29	− 2.93 to − 1.76	− 7.81	< 0.001
Whether subjects observed the trigger event (binomial)	0.93	0.13	0.68 to 1.19	7.14	**< 0.001**
Species (Red ruffed lemur)	− 0.39	0.17	− 0.74 to − 0.05	− 2.26	**0.023**
Behavior (Yawning)	− 1.11	0.27	− 1.65 to − 0.58	− 4.09	**< 0.001**
Model’s centrality	0.40	0.30	− 0.19 to 1.01	1.337	0.182
*Subject’s sex (male)*	0.08	0.14	− 0.20 to 0.36	0.56	0.571
*Distance*	− 0.17	0.06	− 0.31 to -0.04	− 2.56	0.010

## Discussion

In line with our Prediction 1, we found that individuals who had observed the trigger yawning or scratching event were more likely to yawn or scratch as compared to individuals who had not seen the trigger event. This result provides the first evidence of behavioral contagion in black-and-white ruffed lemurs and in red ruffed lemurs. Moreover, it confirms recent findings on behavioral contagion in other Strepsirrhines showing evidence of contagious yawning in wild indri lemurs^3^. Our study further extends this previous work^3^ by showing that also scratching, beside yawning, can elicit behavioral contagion in Strepsirrhines.

Previous studies on semi-free ranging ring-tailed lemurs and red ruffed lemurs had found no evidence of contagious yawning, leading authors to hypothesize that behavioral contagion might be absent or extremely scant in Strepsirrhines^13^. In their study, however, researchers used a different approach to test contagious yawning, by showing individuals videos of conspecifics yawning^13^, rather than observing their reaction to yawning stimuli naturally occurring in the group. Although this approach has been successfully used in some species (e.g., chimpazees^27,28^, bonobos^26^, rhesus macaques, *Macaca mulatta*^33^), it is possible that natural events might be more effective stimuli than videos to trigger behavioral contagion, especially if certain species have different sensitivity to video stimuli. Further studies are therefore needed to assess the response of ring-tailed lemurs when exposed to natural stimuli, as they might also show behavioral contagion when exposed to naturally occurring stimuli of conspecifics.

Our findings suggest that behavioral contagion was already present not only in the common ancestor of Catarrhines and Platyrrhines^25^, but also before they evolutionary split from Strepsirrhines^15,67^. Still, for some primate species, there is yet no evidence of behavioral contagion^13,32^. Future studies will have to better assess whether this lack of evidence depends on methodological constraints (e.g. use of videos rather than natural stimuli, low sample size) or really reflects the fact that behavioral contagion, despite having evolved several million years ago in primates, may only be common in species with more complex social behavior, as some researchers suggest^12,13^. For example, the majority of lemur species are solitary or pair-living^68^, and do not often engage in cooperative behavior^13^. Therefore, according to some authors, these species should be less likely to show behavioral contagion^3^, as this would become common as a result of selection in very social species, but not in others^13^. Both ruffed lemurs and indri lemurs are characterized by high levels of fission-fusion dynamics^3,69,70^, show behavioral synchronization through vocal coordination, and form duets and choruses within the group^71^. In lemurs, this behavior might have emerged with the shift of nocturnal to diurnal activities, which would require more cooperation among individuals, for instance, to reduce the predation risk^3,72^ and increase social cohesion^4^. In ruffed lemur species, behavioral contagion may be especially important as it may drive an adjustment of physiological states and circadian rhythms to allow individuals to quickly align with other group members upon fusions^15,73^, promoting activity synchronization and increasing group coordination^3^. Therefore, future studies will be crucial to assess whether the social organization of a species really determines the presence or absence of behavioral contagion, and more studies in Strepsirrhines will be crucial to this aim.

Although the interaction between behavior and probability of observing the trigger behavior was not significant (i.e. implying that behavioral contagion was similar after observing yawning or scratching events), subjects were more likely to show scratching after a scratching trigger event, than yawning after a yawning trigger event (regardless of whether they had observed the trigger event). These findings can be interpreted in at least two ways. First, it is possible that scratching simply occurs more frequently than yawning, in these species, and thus it is more likely that scratching rather than yawning events will happen within a 2-minute time window. Indeed, this is in line with research in orangutans^39^, Tibetan macaques^38^ and spider monkeys^15^. Second, it is possible that scratching, as compared to yawning, is more likely to occur as a response to similar environmental conditions, so that (regardless of whether subjects observe the trigger event) subjects are more likely to scratch than yawn, after a trigger event, simply because they are more sensitive to the environmental conditions that also cause the initial scratching trigger event. Scratching, for instance, often occurs in contexts of arousal (e.g. post-conflict interactions^74^), which are likely to be perceived by most group members and might generally increase the occurrence of scratching in the group (in contrast to yawning that might be triggered by more individual-specific needs and conditions, like thermoregulation)^5,9^. Similarly, the interaction between species and probability of observing the trigger behavior was not significant (i.e. implying that behavioral contagion was similar in both species), but black-and-white ruffed lemurs were overall more likely than red ruffed lemurs to show scratching or yawning after the corresponding trigger event (regardless of having observed it). As for behavior, it is therefore possible that the probability of engaging in scratching and yawning was simply higher in the black-and-white rather than in the red ruffed lemurs, and/or that the former is more sensitive than the latter to the environmental conditions that trigger these behaviors, so that they are overall more likely to occur in one species, independently of having been observed before.

In contrast to Prediction 2, we found no evidence of a modulating effect of models’ social integration on the probability that subjects would show behavioral contagion. Although we are not aware of previous studies testing the modulating role of model’s social integration for the occurrence of behavioral contagion, these results seem in line with work on bonobos showing no modulating effect of model’s characteristics like rank on the probability of behavioral contagion^63^. In our study, it is possible that the relevance of models’ social integration might have been masked by the fact that all the individuals in the same group were close kin, and thus likely highly relevant to each other. In wild groups, where individuals are not always kin and the group size is frequently larger, results might differ. In the future, it will be interesting to observe larger groups and further assess whether specific characteristics of the model may facilitate behavioral contagion also in Strepsirrhines and in family groups, including in wild individuals.

Our model also revealed a significant effect of the control variable distance, with individuals being closer to the model being more likely to show the same behavior in the following 2 min, regardless of having observed the trigger event. These results are easily explained by considering that individuals that are spatially closer to each other are more likely than distant individuals to experience the same social and ecological conditions (e.g. social uncertainty, light, noise, wind blow)^15,75^, and might thus more likely show the same behavior (e.g. yawning) as a result of the same external factors. Alternatively, it is possible that, when being closer, subjects were more likely to show the same behavior as the model (regardless of having observed the trigger event), because physical proximity might have allowed the detection of other cues (e.g. auditory cues) that can also trigger yawning and scratching contagion, as shown in humans^42,76^ and wild geladas^77^. In the future it will be important to better disentangle between these possible explanations. In any case, the inclusion of distance as a control in the model can be very important: in our case, even if spatially closer individuals were generally more likely to behave in the same way than further individuals, the probability of showing the same behavior was also higher for subjects that observed rather than did not observe the trigger event, regardless of their distance, and this effect was clearly stronger than the one that distance had (see estimates in Table 2).

Overall, our study successfully identified behavioral contagion in both captive black-and-white and red ruffed lemurs, providing support to the hypothesis that behavioral contagion emerged before Strepsirrhines split from Catarrhines and Platyrrhines. Future work should ideally address several other aspects that we could not consider in this study, including the use of trigger events in different modalities (e.g. using auditory cues), the inclusion of models with a larger variety of social characteristics that might affect their relevance in the group, and more detailed analyses (e.g. including the exact latency between the trigger event and the occurrence of the same behavior in other group members). In addition, it might be important to investigate whether the occurrence of aggressive behaviours in the group might affect how subjects respond to yawning and scratching trigger events, as behavioral contagion might be especially relevant to promote social coordination during agonistic interactions to investigate if aggressive behaviors can play a role in the number of displayed trigger events (e.g. scratching and yawning) and if the subject responses following these behaviors would be different from those without the effect of aggressive behaviors. The inclusion of more species with different social characteristics will further allow to understand whether the presence of behavioral contagion is linked to the social complexity of the study species, and to better disentangle the several factors that might modulate this complex phenomenon.

## Electronic Supplementary Material

Below is the link to the electronic supplementary material.

Supplementary Material 1

## Data Availability

Data will be made available upon reasonable request to the first author.
