# Supplementary material for: Contagious yawning and scratching in captive lemurs
Source: Sci Rep. 2024 Nov 4;14:26672. doi: 10.1038/s41598-024-77805-9 (PMC11535043; doi:10.1038/s41598-024-77805-9)
Supplement: Supplementary file 1 — Supplementary Material 1 [file 41598_2024_77805_MOESM1_ESM.rtf]

Contagious yawning and scratching in captive lemurs – Supplementary Material

To explore whether agonistic interactions were linked to an increase in spontaneous and contagious yawning and scratching, and test whether behavioural contagion might be especially relevant to promote social coordination during agonistic interactions, we run the following analyses. Separately for scratching and yawning, we used Spearman exact correlation tests to assess whether, within each 15-minute block of observations, the number of aggressive interactions was linked to the number of trigger events, the number of group members' yawning/scratching events produced after observing the model, or not having observed the model. 
We found a weak but significant positive correlation between the number of trigger events and the number of aggressive interactions, for both yawning (rho: 0.52, N=363, p<0.001) and scratching (rho: 0.14, N=363, p=0.006), which suggests that, when there were aggressive interactions, the number of “spontaneous” yawning and scratching events increased, as expected given that arousal likely increased. 
Moreover, there was a very weak but significant correlation between the number of aggressive interactions and the number of scratching events produced by other group members who had not observed the trigger event (rho: 0.11, N=363, p=0.037). All the other correlations were not significant (i.e. number of aggressive interactions with number of scratching events produced by other group members who had observed the trigger event: rho: 0.08, N=363, p=0.114; with number of yawning events produced by other group members who had not observed the trigger event: rho: -0.03, N=363, p=1.000; with number of yawning events produced by other group members who had observed the trigger event: rho: 0.05, N=363, p=0.347).
These results seem to suggest that yawning and scratching generally increase in frequency in case of aggressive interactions, whereas there is no increase in the frequency of yawning and scratching behaviors produced as a result of behavioural contagion (i.e. after group members observed the original trigger event). Although one could interpret it as preliminary evidence that behavioral contagion fails to serve the function of increasing social cohesion, this interpretation should be taken with caution. In particular, it is likely that social cohesion (and thus, possibly, behavioral contagion) changes in different ways within the group during aggressive interactions, depending on the exact details of the aggression (e.g. increasing between aggressors or victims, but decreasing between victims and aggressors). Unfortunately, our data did not allow us to pursue this level of analysis, but it is surely something that deserves further investigation.  
